# The Influence of Average Temperature and Relative Humidity on New Cases of COVID-19: Time-Series Analysis

**DOI:** 10.2196/20495

**Published:** 2021-01-25

**Authors:** Zonglin He, Yiqiao Chin, Shinning Yu, Jian Huang, Casper J P Zhang, Ke Zhu, Nima Azarakhsh, Jie Sheng, Yi He, Pallavi Jayavanth, Qian Liu, Babatunde O Akinwunmi, Wai-Kit Ming

**Affiliations:** 1 School of Medicine Jinan University Guangzhou China; 2 Faculty of Medicine International School Jinan University Guangzhou China; 3 MRC Centre for Environment and Health Department of Epidemiology and Biostatistics, School of Public Health Imperial College London London United Kingdom; 4 School of Public Health Li Ka Shing Faculty of Medicine The University of Hong Kong Hong Kong Hong Kong; 5 International School Jinan University Guangzhou China; 6 College of Economics Jinan University Guangzhou China; 7 Department of Statistics University of Oxford Oxford United Kingdom; 8 National Media Experimental Teaching Demonstration Center School of Journalism and Communication Jinan University Guangzhou China; 9 Brigham and Women’s Hospital Harvard Medical School Boston, MA United States

**Keywords:** COVID-19, coronavirus, meteorological factors, temperature, humidity, weather, transmission, virus, Asia, time-series, analysis, public health

## Abstract

**Background:**

The influence of meteorological factors on the transmission and spread of COVID-19 is of interest and has not been investigated.

**Objective:**

This study aimed to investigate the associations between meteorological factors and the daily number of new cases of COVID-19 in 9 Asian cities.

**Methods:**

Pearson correlation and generalized additive modeling (GAM) were performed to assess the relationships between daily new COVID-19 cases and meteorological factors (daily average temperature and relative humidity) with the most updated data currently available.

**Results:**

The Pearson correlation showed that daily new confirmed cases of COVID-19 were more correlated with the average temperature than with relative humidity. Daily new confirmed cases were negatively correlated with the average temperature in Beijing (*r*=–0.565, *P*<.001), Shanghai (*r*=–0.47, *P*<.001), and Guangzhou (*r*=–0.53, *P*<.001). In Japan, however, a positive correlation was observed (*r*=0.416, *P*<.001). In most of the cities (Shanghai, Guangzhou, Hong Kong, Seoul, Tokyo, and Kuala Lumpur), GAM analysis showed the number of daily new confirmed cases to be positively associated with both average temperature and relative humidity, especially using lagged 3D modeling where the positive influence of temperature on daily new confirmed cases was discerned in 5 cities (exceptions: Beijing, Wuhan, Korea, and Malaysia). Moreover, the sensitivity analysis showed, by incorporating the city grade and public health measures into the model, that higher temperatures can increase daily new case numbers (beta=0.073, Z=11.594, *P*<.001) in the lagged 3-day model.

**Conclusions:**

The findings suggest that increased temperature yield increases in daily new cases of COVID-19. Hence, large-scale public health measures and expanded regional research are still required until a vaccine becomes widely available and herd immunity is established.

## Introduction

In December 2019, several cases of pneumonia of unknown etiology were reported in Wuhan, Hubei Province, China [1]. A novel strain of coronavirus was identified from the nasopharyngeal swab specimens of infected patients, which was later named SARS-CoV-2, which results in the disease COVID-19. As the number of infectees increased, the World Health Organization declared the outbreak as a public health emergency of international concern on January 31, 2020 [2].

SARS-CoV-2, of the *Coronaviridae* family, is an enveloped, single-stranded, positive-sense RNA (ribonucleic acid) virus, which is closely related to the SARS (severe acute respiratory syndrome)-like coronaviruses, and based on the phylogenetic analysis, these coronaviruses have a common ancestor that resembles the bat coronavirus HKU9-1 [3,4]. Evidence has shown that SARS-CoV-2 can transmit from person to person via respiratory droplets, fecal-oral route, direct contact, and aerosols [5-7]. Moreover, the long incubation period (1-14 days) increases the difficulty of controlling the COVID-19 outbreak. Studies have shown the mean incubation period to be 5.1 days (range: 2.2-11.5 days, 95% CI 4.5-5.8) [8]; one study estimated the mean incubation period to be 6.4 days (range: 2-14 days) [9]. By early July 2020, 215 countries and regions had reported high infection rates, with over 7,000,000 confirmed cases, 400,000 deaths, and a fatality rate of over 5.84% worldwide [10].

Human respiratory pathogens (bacterial pathogens like pneumococcus and viruses like rubella and influenza) usually exhibit an annual, seasonal pattern, with an increase in incidence during winter and a decrease during summer. Although there is much data on the influenza virus, respiratory syncytial virus, and the SARS outbreak in 2003 following this pattern, it is difficult to predict whether COVID-19 will follow the trend and be eliminated during warmer seasons, since our understanding of the forces driving the seasonality of infectious diseases remains limited. As influenza is a common viral disease, a proportion of the population already possesses some levels of immunity, and when more patients recover, herd immunity constrains the transmission of the virus. The low or absent prevalence of SARS-CoV or the Middle East respiratory syndrome coronavirus (MERS-CoV) in the summer was also observed to have strongly relied on the use of effective therapeutic treatment and strict public health measures [11,12]. However, SARS-CoV-2 is a novel virus among humans, immunity to this ongoing viral pandemic is limited, and an effective pharmaceutical therapy or vaccine is yet to be available.

Seasonality in the outbreak of respiratory infectious diseases may be due to seasonal variations in host physiology (susceptibility, individual immunity, and herd immunity) [13], genetics [14], viral stability [15,16] and infectivity [17-20], presence of latent infectors to spread the virus [21,22], and atmospheric dispersion and transocean intercontinental migration [23-26], which are mainly driven by meteorological factors, including temperature and humidity [17]. Geological features and latitude play a major role in forming a meteorological pattern. Lowen et al [19] assumed the seasonality of influenza and respiratory syncytial virus epidemics in temperate climates were mainly attributable to the low absolute humidity, and specific factors associated with it, namely low temperature, increased population, and low micronutrient levels (such as low vitamin D levels) [27,28]. During the winter in temperate zones, temperature and humidity are low, there is dryness and coldness, and viruses are more easily transmitted via aerosols than direct or indirect contact, where the low temperature renders the virus viable and stable in aerosols and on surfaces. However, in the rainy seasons of tropical regions, where it is hot and wet, aerosol transmission decreases but transmission by direct contact increases [29]. Although high temperatures can decrease the stability of the virus and reduce the level of aerosolization of viral droplets, the amount of virus deposited on surfaces increases as the temperature increases [30].

Because of the relatively stable structure of the coronaviruses, the infectivity of the coronavirus would not be affected by a relative humidity >95% and a temperature of 28 °C to 33 °C [30]. For example, the transmission of SARS is more efficient in temperatures between 16 °C and 28 °C, and the wide use of air-conditioning also provides shelter for the breeding and transmission of SARS, where the virus is stable for 3 weeks at room temperature [30]. SARS-CoV-2 has many similarities with SARS-CoV-1, but whether meteorological factors influence viral transmission has not been established. Therefore, in this study, we investigated whether and how meteorological factors affect the spread of COVID-19, with the specific aim of examining the relationship between meteorological factors and the number of COVID-19 daily cases in Asian cities at different latitudes. The objective was to provide scientific evidence concerning the future progression of COVID-19 based on climate factors.

## Methods

### Meteorological Data

We obtained daily meteorological data from the National Meteorological Information Center [31]. The meteorological data of cities in China were collected from the National Meteorological Bureau and Data Center of the Ministry of Ecology [32,33] from January 20 to March 18, 2020. The meteorological data in Seoul, Tokyo, Kuala Lumpur, and Singapore were obtained from timeanddate.com [34] from January 20 to March 18, 2020. We retrieved the highest and lowest temperatures and relative humidity for four different quarters of the day and computed the average values of temperature and relative humidity for that day.

### COVID-19 Surveillance Data

The daily number of domestic COVID-19 cases in 5 cities in China (Wuhan, Beijing, Shanghai, Guangzhou, and Hong Kong) and Singapore were obtained from the Ministry of Health in China [35] and Singapore [36], respectively, from the inception of cases to March 18, 2020. Given that the daily number of domestic cases at the city level in Japan, Korea, and Malaysia were not available from their corresponding Ministries of Health, as an alternative, we used the number of domestic cases at the national level in these 3 countries for analysis, which was obtained from the Ministry of Health of Japan, Korea, and Malaysia, respectively. The clinical criterion for the diagnosis of COVID-19 was based on the high-throughput sequencing or reverse transcription polymerase chain reaction assay via nasopharyngeal swab.

### Statistical Analysis

First, the normality of the daily new cases and meteorological data was evaluated by examining their skewness and kurtosis. Subsequently, descriptive analysis was performed to analyze the city-specific characteristics of confirmed COVID-19 cases. Second, to analyze the overall correlation between the two meteorological factors and daily new confirmed cases, Pearson correlation and covariances between the daily COVID-19 new cases and daily meteorological factors were tested, and the linearity between variables was tested using linear regression (with city level and public health measures as controlling factors), as shown in Multimedia Appendix 1. All statistical analyses were performed using STATA 14.0 (Stata Corp).

Subsequently, city-specific generalized additive models (GAMs) with a Poisson family and logarithm link function were used to estimate the associations of daily COVID-19 new cases with average temperature and relative humidity. GAMs are useful for identifying exposure-response relationships from various types of data, particularly in exploring nonparametric relationships [37]. The model was built as follows:

Daily new cases_i_ ~ Poisson (μ_i_)

*log μ_i_ =* β_0_ + *f*_1_ *(Date_i_)* + *f*_2_ *(Temperature_i_)* + *f*_3_ *(Relative humidity_i_)*

where the terms *f_1_* to *f_2_* are the smoothing functions, and *β_0_* is the intercept. The GAM analysis was performed in R software, version 3.6.0 (The R Project for Statistical Computing), using the package “mgcv.” We first established a basic temporal model for COVID-19 cases without including meteorological variables. To adjust for long-term trends and seasonality, we included penalized spline functions of time in the model. The degrees of freedom (df) for time was optimized by minimizing the absolute values of the partial autocorrelation function (PACF) of residuals for lags up to 30 days [38-41]. Additionally, the selection of an optimal model was based on the lowest Akaike information criterion (AIC). Next, we built meteorological models based on the temporal models to account for the lagged effect of meteorological variables and the incubation period of COVID-19 [7,42]. Specifically, we examined the effect of meteorological variables with different time lags, including 1-day lag (lag 1d), 3-day lag (lag 3d), 5-day lag (lag 5d), single-week lag (lag 7d), and 2-week lag (lag 14d), to capture immediate effects and lagged effects, respectively. Automated penalized splines were used to fit the association between the daily new cases and each of the meteorological variables. The date when the accumulated cases exceeded 30 in each city or country was selected as the inception of the date incorporated in the model to equalize the starting speed of outbreak and thus avoid misinterpretation and overfitting.

To control for the autocorrelation, the model’s residuals were examined for serial correlation using PACF. Default plots showing the smooth components of a fitted GAM were produced. The percentage of deviance explained by each variable was calculated, which represents the scale of a linear predictor that contributes to the component smooth functions. At last, the graph of smooth components was grouped based on similar latitudes to mitigate the influence by latitude.

### Sensitivity Analysis

To verify model results, a sensitivity analysis was performed. Owing to the different public health measures put forward in different cities or countries, the city scale was taken into consideration in the model. We postulated that the during the following 15 days after the accumulated cases exceeded 40, public health measures and precautions were not readily available; thus, the transmission of the disease resembles natural transmission. The model was built as follows:

*Daily new cases_i_* ~ *Poisson (μ_i_)*

*log μ_i_ =* β_0_ + *f_1_* *(Date_i_)* + *f*_2_ *(Temperature_i_)* + *f*_3_ *(Relative humidity_i_)* + *f*_4_ *(City level_i_)*

where the terms *f_1_* is the smoothing functions, *f_2-4_* are the linear functions, and *β_0_* is the intercept. GAM with linear components facilitate the analysis of the effects of explanatory variables in a way that closely resembles the analysis of covariates in a standard linear model, but with less confining assumptions. This is achieved by specifying a link function, which links the systematic component of the linear model with a wider class of outcome variables and residual forms.

## Results

### Descriptive Statistical Results

The daily new cases of COVID-19 and meteorological data from January 20 to March 18, 2020 (61 days), in 9 different cities are shown in Tables 1 and 2. During the study period, the temperature in the 5 cities in China ranged from –9 °C to 26.25 °C (Beijing: –9 °C to 26 °C; Shanghai: 0 °C to 22 °C; Guangzhou 8.75 °C to 26.25 °C; Wuhan: 1 °C to 21.75 °C; Hong Kong: 11.25 °C to 26.25 °C). The temperature in Singapore ranged from 25.5 °C to 32.75 °C; in Seoul, from –9.5 °C to 12.25 °C; in Tokyo, from 0.75 °C to 18.5 °C; and in Kuala Lumpur, from 24.5 °C to 33.5 °C. Multimedia Appendices 2 and 3 show the distribution of daily COVID-19 new cases associated with average temperature and average relative humidity over time, respectively.

**Table 1:** Descriptive statistics for different Asian cities.

Variables (latitude)		Mean (SD)	Minimum	Q1	Median	Q3	Maximum
**Beijing, China (39°56′N)**							
	Domestic case (persons)	8.00 (9.60)	0.00	1.00	5.00	12.00	39.00
	Highest temperature (°C)	8.29 (5.34)	–4.00	5.00	8.00	11.00	26.00
	Lowest temperature (°C)	–3.10 (3.44)	–9.00	–6.00	–3.00	–1.00	4.00
	Relative humidity (%)	52.49 (19.48)	23.50	36.25	54.75	67.00	92.75
**Shanghai, China (31°14′N)**							
	Domestic case (persons)	7.00 (8.20)	0.00	0.00	3.00	13.00	27.00
	Highest temperature (°C)	11.34 (3.61)	4.00	8.75	10.75	13.50	21.50
	Lowest temperature (°C)	8.28 (3.18)	1.50	6.25	8.25	10.25	15.00
	Relative humidity (%)	71.11 (14.70)	42.00	60.00	71.75	82.50	94.75
**Wuhan, China (30°37′N)**							
	Domestic case (persons)	1035.00 (1968.80)	0.00	120.00	502.50	1524.50	13,436.00
	Highest temperature (°C)	11.45 (4.40)	3.50	8.00	11.00	14.00	20.75
	Lowest temperature (°C)	8.22 (3.76)	1.50	5.25	8.25	11.00	17.50
	Relative humidity (%)	74.03 (11.70)	51.75	66.25	74.75	84.75	93.25
**Guangzhou, China (23°10′N)**							
	Domestic case (persons)	7.00 (9.80)	0.00	0.00	3.00	12.00	38.00
	Highest temperature (°C)	19.32 (3.95)	11.25	16.25	20.00	22.00	26.25
	Lowest temperature (°C)	16.55 (3.82)	8.75	13.75	17.00	19.75	22.50
	Relative humidity (%)	67.95 (13.19)	34.75	60.00	68.75	78.50	87.50
**Hong Kong, China (22°15′N)**							
	Domestic case (persons)	2.00 (2.50)	0.00	0.00	1.50	3.00	13.00
	Highest temperature (°C)	20.66 (3.11)	13.50	18.50	21.00	23.25	26.25
	Lowest temperature (°C)	18.39 (3.12)	11.25	16.50	19.25	20.50	24.25
	Relative humidity (%)	70.31 (12.67)	31.00	65.00	74.00	79.00	88.50

**Table 2:** Descriptive statistics for different Asian cities, continued.

Variables (latitude)		Mean (SD)	Minimum	Q1	Median	Q3	Maximum
**Singapore (1°18′N)**							
	Domestic case (persons)	3.00 (3.10)	0.00	1.00	3.00	4.00	14.00
	Highest temperature (°C)	30.46 (0.97)	27.75	29.75	30.50	31.25	32.75
	Lowest temperature (°C)	27.64 (0.77)	26.00	27.25	27.75	28.25	29.00
	Relative humidity (%)	76.09 (4.95)	63.75	73.25	75.50	79.25	89.50
**Seoul, Korea (37°33′N)**							
	Domestic case (persons)	142.00 (216.00)	0.00	0.00	3.00	210.00	909.00
	Highest temperature (°C)	5.45 (3.93)	–6.00	3.25	6.25	8.00	12.25
	Lowest temperature (°C)	0.95 (3.56)	–9.50	–1.25	1.50	3.75	6.25
	Relative humidity (%)	68.50 (10.78)	48.25	59.50	66.75	76.75	91.50
**Tokyo, Japan (35°69′N)**							
	Domestic case (persons)	15.00 (17.90)	0.00	0.00	8.00	25.00	60.00
	Highest temperature (°C)	10.30 (3.11)	3.50	8.75	10.25	12.50	18.50
	Lowest temperature (°C)	6.92 (2.65)	0.75	5.25	6.75	8.75	14.00
	Relative humidity (%)	61.41 (17.52)	32.75	47.25	59.50	76.25	97.75
**Kuala Lumpur, Malaysia (3°8′N)**							
	Domestic case (persons)	14.00 (37.20)	0.00	0.00	0.00	7.00	190.00
	Highest temperature (°C)	30.98 (1.26)	26.50	30.25	31.00	31.75	33.50
	Lowest temperature (°C)	27.19 (1.03)	24.50	26.50	27.25	28.00	29.00
	Relative humidity (%)	77.19 (6.74)	62.50	74.00	77.50	82.25	92.50

To test the potential collinearity between the meteorological parameters, a series of Pearson correlations and covariances, as well as linear regressions were conducted (Table 3 and Multimedia Appendix 1). Daily confirmed new cases were negatively correlated with average temperature in Beijing (*r*=–0.565, *P*<.001), Shanghai (*r*=–0.471, *P*<.001), and Guangzhou (*r*=–0.530, *P*<.001). In contrast, Japan exhibited a positive correlation (*r*=0.416, *P*<.001). The correlation between average temperature and relative humidity was found to be positive in Shanghai, Guangzhou, Hong Kong, Korea, and Japan, and negative in Beijing, Wuhan, Singapore, and Malaysia, according to the pairwise Pearson correlation test (Table 3).

**Table 3:** Pearson correlation coefficient (*r*) between daily new COVID-19 cases and meteorological factors.

Variables			Daily new cases					Average temperature		
			*r*		*P* value		*r*			*P* value
**Beijing, China**										
	Daily new cases	1.00		—^a^		—			—	
	Average temperature	–0.57		<.001		1.00			—	
	Humidity	0.15		.30		–0.26			.05	
**Shanghai, China**										
	Daily new cases	1.00		—		—			—	
	Average temperature	–0.47		<.001		1.00			—	
	Humidity	–0.11		.45		0.09			.50	
**Wuhan, China**										
	Daily new cases	1.00		—		—			—	
	Average temperature	0.04		.81		1.00			—	
	Humidity	0.10		.48		–0.42			<.001	
**Guangzhou, China**										
	Daily new cases	1.00		—		—			—	
	Average temperature	–0.53		<.001		1.00			—	
	Humidity	–0.29		.05		0.34			.01	
**Hong Kong, China**										
	Daily new cases	1.00		—		—			—	
	Average temperature	0.08		.56		1.00			—	
	Humidity	0.07		.60		0.58			<.001	
**Singapore**										
	Daily new cases	1.00		—		—			—	
	Average temperature	0.27		.04		1.00			—	
	Humidity	0.04		.74		–0.58			<.001	
**Seoul, Korea**										
	Daily new cases	1.00		—		—			—	
	Average temperature	0.29		.02		1.00			—	
	Humidity	0.14		.30		0.32			.01	
**Tokyo, Japan**										
	Daily new cases	1.00		—		—			—	
	Average temperature	0.42		<.001		1.00			—	
	Humidity	0.20		.14		0.21			.11	
**Kuala Lumpur, Malaysia**										
	Daily new cases	1.00		—		—			—	
	Average temperature	0.21		.11		1.00			—	
	Humidity	–0.17		.20		–0.74			<.001	

^a^Not applicable.

### City-Specific GAM Analysis of Daily New COVID-19 Cases With Meteorological Factors

The final GAM model of daily new COVID-19 cases incorporated date (time-series), average temperature, and mean relative humidity. All estimates and significance levels were listed in Multimedia Appendix 1. The models with the best performance (lowest AIC) for each city were as follows: no-lag model for Shanghai and Singapore; lag 1d model for Beijing and Wuhan; lag 5d model for Guangzhou, Korea, and Kuala Lumpur; and lag 14d model for Hong Kong and Japan (Table 4).

GAM results were reported using the smoothing components plot for temperature and relative humidity in Multimedia Appendices 4 and 5, respectively. The smoothing components plots demonstrated the estimated smoothing spline functions with the linear effect subtracted out, and each panel represented the weighted sum of basis functions for each time-varying covariate and corresponds to the hypothesized model.

The significant smoothers indicated that the correlations between new cases of COVID-19 and explanatory variables were nonlinear. As shown in Multimedia Appendix 4, the no-lag model suggested that holding all linear and the other nonlinear terms fixed, daily new cases of COVID-19 were not influenced by the temperature, but the case number decreased when the temperature reached 5 °C, 18 °C, and 29 °C in Beijing, Hong Kong, and Singapore, respectively (*P*<.01 for all; Multimedia Appendix 4). While the magnitude of the results may look small relative to the base rate of case accrual, these plots were on the log-case scale, so the effect on the case number is multiplicative. The distributions of COVID-19 cases displayed greater uncertainty at a lower temperature in Beijing and Wuhan. Beijing and Wuhan have fluctuating patterns throughout the 6 models both regarding temperature and relative humidity except in the lag 5d and lag 4d models for Wuhan, which may be due to the change in diagnostic method on February 12, when a total of 13,436 cases were added. Nevertheless, the relationship between relative humidity and new cases were less evident in the no-lag model, and the distributions of COVID-19 cases displayed greater uncertainty in Wuhan.

**Table 4:** The selection of generalized additive modeling by Akaike information criterion (AIC).

City and model		*df*	AIC
**Beijing, China**			
	No lag	24.01	265.47
	Lag 1d^a^	21.71	226.47
	Lag 3d	17.72	273.59
	Lag 5d	21.46	263.25
	Lag 7d	23.38	243.26
	Lag 14	19.79	230.50
**Shanghai, China**			
	No lag^a^	7.91	178.01
	Lag 1d	7.50	181.60
	Lag 3d	7.04	180.93
	Lag 5d	7.08	180.60
	Lag 7d	6.83	182.73
	Lag 14	10.51	178.06
**Guangzhou, China**			
	No lag	14.17	199.85
	Lag 1d	14.40	192.44
	Lag 3d	18.32	194.49
	Lag 5d^a^	20.97	192.35
	Lag 7d	10.63	200.94
	Lag 14	12.89	195.34
**Wuhan, China**			
	No lag	27.95	4812.57
	Lag 1d^a^	27.87	3637.25
	Lag 3d	27.89	6778.17
	Lag 5d	27.98	5344.86
	Lag 7d	27.86	10,351.23
	Lag 14	26.45	6735.71
**Hong Kong, China**			
	No lag	12.46	193.98
	Lag 1d	16.49	192.14
	Lag 3d	11.92	195.65
	Lag 5d	14.12	193.45
	Lag 7d	13.17	199.01
	Lag 14^a^	6.53	189.09
**Singapore**			
	No lag^a^	10.94	183.22
	Lag 1d	10.88	185.19
	Lag 3d	9.57	202.13
	Lag 5d	10.83	207.79
	Lag 7d	16.18	230.09
	Lag 14	14.83	277.87
**Seoul, Korea**			
	No lag	27.54	461.16
	Lag 1d	25.86	381.98
	Lag 3d	27.19	439.14
	Lag 5d^a^	26.67	363.42
	Lag 7d	26.24	420.27
	Lag 14	27.29	452.58
**Tokyo, Japan**			
	No lag	24.58	264.80
	Lag 1d	19.77	266.44
	Lag 3d	24.37	276.50
	Lag 5d	18.28	272.25
	Lag 7d	17.61	288.62
	Lag 14^a^	18.33	264.45
**Kuala Lumpur, Malaysia**			
	No lag	16.37	142.50
	Lag 1d	16.74	138.72
	Lag 3d	21.70	132.95
	Lag 5d^a^	17.93	125.05
	Lag 7d	19.80	132.67
	Lag 14	21.58	133.89

^a^Denotes the model with the lowest AIC value.

### Sensitivity Analysis of Daily New COVID-19 Cases With Meteorological Factors

After taking the city level into consideration in the model, a GAM model of daily new COVID-19 cases was built, incorporating date (time-series), city level, average temperature, and mean relative humidity, where the date was analyzed as spline functions.

Overall, we found a significant association between meteorological factors and daily new cases of COVID-19 in 9 Asian cities. Under GAM, high temperature tended to increase the number of daily new cases of COVID-19 whereas high relative humidity decreased the count (Table 5). Relative humidity does not influence the daily new cases significantly, but higher temperatures can exert an increase as high as 7% on daily new cases in lag 3d (beta=0.073, SE 0.006, *P*<.001; Table 4). The time-series analysis revealed that notwithstanding the lagged time effects, the overall influence of temperature was steadily positive. Nevertheless, the relative humidity exerted a negative influence on the transmission of daily new cases of COVID-19 (Table 5).

**Table 5:** Generalized linear modeling of the effects of temperature and relative humidity on daily new cases of COVID-19.

Model		Estimate	Standard error	Z value	*P* value
**No-lag model**					
	Temperature	0.062	0.006	10.861	<.001
	Relative humidity	–0.018	0.001	–15.105	<.001
**Lag 1d model**					
	Temperature	0.015	0.007	2.284	.02
	Relative humidity	–0.007	0.001	–6.513	<.001
**Lag 3d model**					
	Temperature	0.073	0.006	11.594	<.001
	Relative humidity	–0.006	0.001	–4.501	<.001
**Lag 5d model**					
	Temperature	–0.026	0.006	–4.152	<.001
	Relative humidity	0.003	0.001	2.471	.01
**Lag 7d model**					
	Temperature	0.063	0.005	13.887	<.001
	Relative humidity	–0.008	0.001	–6.620	<.001

## Discussion

### Principal Findings

In this study, we investigated the associations between meteorological factors and patterns of daily new cases of COVID-19 across 9 Asian cities. The city-specific GAM analysis revealed a positive relationship between temperature and daily new cases of COVID-19 in Guangzhou, Singapore (except in the lagged 14-day model), Hong Kong (except in the lagged 7-day and 14-day model), and Beijing (high curvilinearity). Relative humidity positively associated with the number of daily new cases in Singapore (except in the lagged 14-day model), Hong Kong (except in the lagged 3-day model). Moreover, the sensitivity test using GAM with linear components revealed that high temperature significantly increases the daily new cases of COVID-19, while high relative humidity significantly reduced, to a lower extent, the daily new cases of COVID-19. Therefore, our analysis suggests, unlike influenza, seasonality of COVID-19 may not be expected, and the pandemic is unlikely to diminish during warmer seasons (ie, summer).

Researchers have long been investigating how meteorological factors affect the viral infectivity, where GAM has been frequently used, as it allows smooth components to be estimated for time, meteorological factors, and other covariates, together with a nonsmoothed period effect. Experiments from the mid-20th century reported that the influenza virus is more stable in cool and dry air [15,16]. With increasing temperature, the viability of the influenza virus in aerosol or droplets [43] and the aerosol transmission diminishes [17]. Lowen et al [19] reported that aerosol transmission of influenza between guinea pigs was completely blocked at temperature higher than 30 °C despite evidence of continuous viral shedding from infectious individuals; nevertheless, direct contact transmission was not affected, which was equally efficient at 30 °C and 20 °C. Chan et al [30] reported that the viability of the SARS virus was rapidly lost (>3 log 10) at high temperatures (38 °C) and high relative humidity (>95%). The better stability of the SARS coronavirus at low temperatures and low humidity environment might facilitate its transmission in the community in subtropical areas (such as Hong Kong) during the spring and in air-conditioned environments. This might also explain why some Asian countries in tropical areas (such as Malaysia, Indonesia, or Thailand) with high temperatures and high relative humidity environment did not have major community outbreaks of SARS. However, such an explanation is not currently convincing in light of the results of the present study, where higher temperatures were associated with increases in the daily new cases of COVID-19 in some of the investigated regions. The high temperature and high relative humidity in tropical Asian countries like Singapore and Malaysia also seem to have little influence on the growing number of daily new cases (Multimedia Appendix 4). However, this could have been confounded by multiple factors.

There are several reasons underlying the continuous growth in COVID-19 cases in Singapore and Malaysia, such as the high population density, mass gatherings, use of air-conditioning, and shortage of medical resources [30]. SARS-CoV-2 can persist at room temperature for up to 9 days, and its heat sensitivity renders it susceptible to increased temperature, affecting its persistence in the outdoor environment. However, this coronavirus was still found to be infective up to 2 weeks in an air-conditioned environment [30]. As air-conditioners may increase the probability of viral spread, it may be advisable to reduce the use of air-conditioners and keep areas well ventilated [44]. Moreover, during low temperature and high humidity, it is advisable to avoid mass gatherings, since there is evidence supporting transmission by direct contact or close contact in tropical areas.

Humidity can influence aerosol transmission by altering the proportion of respiratory droplets undergoing aerosolization and influencing the stability and viability of the virus within these aerosols. Respiratory droplets are generated in the high humidity setting of the respiratory tract. Upon entering an environment with low humidity, respiratory droplets reduce in size within seconds due to evaporation. At higher environmental humidity, respiratory droplets evaporate more slowly, and hence are larger and settle faster, and less aerosol nuclei are produced [45,46]. Previous studies have shown that influenza transmission in mice decreased as relative humidity increased from 47% to 70% [13,47]. Moreover, humidity can also influence indirect transmission by changing the mass of respiratory droplets accumulating on surfaces and affecting the survival of the virus on surfaces. While increased humidity reduces the number of droplet nuclei formed, the same mechanisms (reduced droplet evaporation and faster droplet settling) result in a greater mass of respiratory droplets on surfaces [45,46]. Areas with relatively low temperature and humidity have a higher infection rate compared to tropical areas since cold and dry weather is suitable for viral survival and transmission [48]. The viability of the influenza virus appears greater at lower humidity, and exhibits progressively reduced survival with increasing relative humidity over the 27%-84% range, with an increase in survival at 99% relative humidity. The mechanisms underlying this may be that the reduced evaporation of droplets at high relative humidity maintain the solute concentration, thus protecting the virus [17,49]. In addition, the 30° N to 50° N latitudes have become a zone for COVID-19 transmission with a similar average temperature between 5 °C and 11 °C and 47%-79% humidity, which may be influenced by the transoceanic migration of the virus, but the underlying mechanism is still not understood [50].

Moreover, under laboratory conditions with constant humidity and temperature, circadian and circannual rhythms in variation of susceptibility of hosts (mice) have been observed [13,51]. Mice were substantially more susceptible to invasive pneumococcal disease in early morning hours than any other time of day [51], and more susceptible to influenza in winter than in summer [13], which was thought to be attributable to the daily and seasonal variation of melatonin [52]. In addition, even in areas where many spend summers in air-conditioned spaces, marked annual variations in incidence constantly exist, and similar strains of the virus appear almost simultaneously across vast stretches of ocean in areas of similar latitude around the globe [23,24].

Therefore, although a higher temperature is associated with lower effectiveness of virus transmission, yet it does not necessarily suggest a reduced chance for virus survival. The natural fading out of the virus in the summer is unlikely given the widespread use of air-conditioners in developed areas and dense populations in cities. Until an effective vaccine becomes widely available for the establishment of herd immunity, alongside efficient pharmaceutical therapies, strict public health measures should be implemented, including social distancing, quarantine, contact tracing, face mask wearing, and hand washing.

### Strengths and Limitations

This is the first study to statistically analyze the relationship between meteorological factors and the daily new cases of COVID-19. Because of the nonlinear nature of the data, we also performed GAMs to quantitate and visualize the relationship using spline functions. However, there are several limitations. First, the number of cases in Malaysia, Korea, and Japan were obtained from the epidemiologic reports released by the Department of Health in the corresponding countries, instead of a daily update of case numbers, which was not available for these countries. Hence, some cases may be missing. Second, the duration of the study period was short and the number of cases in Malaysia, Singapore, and Japan were small. Third, we only considered two meteorological factors (temperature and humidity) in this study. Other covariates such as wind speed, pollutant concentration, population density, air-conditioning use, and rainfall, which could also influence the spread of COVID-19, were not included. Moreover, while we collected the meteorological data from the capital cities of Malaysia, Japan, and Korea, the number of domestic cases at the national level in these three countries was used for analysis due to lack of available data at their city level. Most importantly, we did not incorporate public health measures into the modeling, which may greatly confound the results, but we chose the date when accumulated confirmed cases exceeded 30, based on the postulation that a certain level of public health measures had been carried out at that time; thus, the confounding effects can be mitigated at some point.

### Conclusions

In this study, we found high temperature to be associated with daily new cases of COVID-19. Therefore, unlike influenza, seasonality in COVID-19 prevalence may not be expected, and the pandemic is unlikely to decrease in numbers during the warmer seasons. Strict public health measures such as social distancing, quarantine, contact tracing, face mask wearing, and hand washing are needed until a vaccine becomes widely available to induce herd immunity.

## Appendix

Multimedia Appendix 1 The effects of temperature and relative humidity on daily new cases of COVID-19.

Multimedia Appendix 2 Daily temperature and distribution of daily new cases of COVID-19 over time.

Multimedia Appendix 3 Daily relative humidity and distribution of daily new COVID-19 cases over time.

Multimedia Appendix 4 Smoothing components plots for daily new COVID-19 cases associated with average temperature.

Multimedia Appendix 5 Smoothing components plots for daily new COVID-19 cases associated with average relative humidity.

## Abbreviations

AIC: Akaike information criterion
df: degrees of freedom
GAM: generalized additive modeling
MERS-CoV: Middle East respiratory syndrome coronavirus
PACF: partial autocorrelation function
RNA: ribonucleic acid
SARS-CoV: severe acute respiratory syndrome coronavirus
SARS: severe acute respiratory syndrome
