# Supplementary material for: The Influence of Average Temperature and Relative Humidity on New Cases of COVID-19: Time-Series Analysis
Source: JMIR Public Health Surveill. 2021 Jan 25;7(1):e20495. doi: 10.2196/20495 (PMC7836910; doi:10.2196/20495)
Supplement: Multimedia Appendix 1 [file publichealth_v7i1e20495_app1.docx]

**Supplementary materials:**

Supplementary Table 1. The effects of temperature and relative humidity on daily new cases of COVID-19

| Variables | | estimate | standard error | P-value for parametric coefficients | edf | Ref.df | P-value for smooth terms | R-squared | UBRE |
| --- | --- | --- | --- | --- | --- | --- | --- | --- | --- |
| **Lag 0d model** | |  |  |  |  |  |  |  |  |
| *Beijing, China* | | 1.22 | 0.19 | 0.00 |  |  |  |  |  |
|  | Temperature |  |  |  | 7.82 | 8.30 | <0.001 | 0.69 | 1.40 |
|  | Relative humidity |  |  |  | 6.26 | 7.64 | 0.00 |  |  |
| *Shanghai, China* | | 0.52 | 0.26 | 0.04 |  |  |  |  |  |
|  | Temperature |  |  |  | 2.12 | 2.69 | 0.11 | 0.93 | 0.02 |
|  | Relative humidity |  |  |  | 1.00 | 1.00 | 0.13 |  |  |
| *Wuhan, China* | | 5.13 | 0.03 | 0.00 |  |  |  |  |  |
|  | Temperature |  |  |  | 8.99 | 9.00 | 0.00 | 0.95 | 69.61 |
|  | Relative humidity |  |  |  | 8.98 | 9.00 | 0.00 |  |  |
| *Guangzhou, China* | | 0.13 | 0.32 | 0.68 |  |  |  |  |  |
|  | Temperature |  |  |  | 1.00 | 1.00 | 0.04 | 0.88 | 0.54 |
|  | Relative humidity |  |  |  | 6.05 | 7.14 | <0.001 |  |  |
| *Hong Kong, China* | | 0.73 | 0.10 | 0.00 |  |  |  |  |  |
|  | Temperature |  |  |  | 3.67 | 4.54 | 0.06 | 0.49 | 0.73 |
|  | Relative humidity |  |  |  | 1.00 | 1.00 | 0.93 |  |  |
| *Singapore* | | 1.26 | 0.08 | 0.00 |  |  |  |  |  |
|  | Temperature |  |  |  | 2.09 | 2.63 | 0.14 | 0.57 | 0.15 |
|  | Relative humidity |  |  |  | 1.32 | 1.56 | 0.72 |  |  |
| *Seoul, Korea* | | 2.09 | 0.30 | 0.00 |  |  |  |  |  |
|  | Temperature |  |  |  | 8.74 | 8.95 | 0.00 | 0.96 | 3.77 |
|  | Relative humidity |  |  |  | 8.84 | 8.99 | 0.00 |  |  |
| *Tokyo, Japan* | | 1.60 | 0.18 | 0.00 |  |  |  |  |  |
|  | Temperature |  |  |  | 7.58 | 8.17 | <0.001 | 0.95 | 0.75 |
|  | Relative humidity |  |  |  | 8.10 | 8.74 | 0.00 |  |  |
| *Kuala lumpur, Malaysia* | | -1.09 | 0.87 | 0.21 |  |  |  |  |  |
|  | Temperature |  |  |  | 5.69 | 6.39 | 0.00 | 1.00 | 0.30 |
|  | Relative humidity |  |  |  | 3.05 | 3.53 | 0.00 |  |  |
| **Lag 1d model** | |  |  |  |  |  |  |  |  |
| *Beijing, China* | | 1.24 | 0.13 | 0.00 |  |  |  |  |  |
|  | Temperature |  |  |  | 8.93 | 8.99 | 0.00 | 0.91 | 0.61 |
|  | Relative humidity |  |  |  | 7.67 | 8.52 | 0.00 |  |  |
| *Shanghai, China* | | 0.57 | 0.25 | 0.02 |  |  |  |  |  |
|  | Temperature |  |  |  | 1.62 | 2.02 | 0.37 | 0.90 | 0.09 |
|  | Relative humidity |  |  |  | 1.00 | 1.00 | 0.86 |  |  |
| *Wuhan, China* | | 4.97 | 0.06 | 0.00 |  |  |  |  |  |
|  | Temperature |  |  |  | 9.00 | 9.00 | 0.00 | 0.97 | 49.84 |
|  | Relative humidity |  |  |  | 8.98 | 9.00 | 0.00 |  |  |
| *Guangzhou, China* | | 0.11 | 0.33 | 0.75 |  |  |  |  |  |
|  | Temperature |  |  |  | 1.34 | 1.58 | 2.17 | 0.92 | 0.40 |
|  | Relative humidity |  |  |  | 7.29 | 8.25 | <0.001 |  |  |
| *Hong Kong, China* | | 0.67 | 0.11 | 0.00 |  |  |  |  |  |
|  | Temperature |  |  |  | 7.71 | 8.53 | 0.02 | 0.61 | 0.69 |
|  | Relative humidity |  |  |  | 1.00 | 1.00 | 0.25 |  |  |
| *Singapore* | | 1.27 | 0.08 | 0.00 |  |  |  |  |  |
|  | Temperature |  |  |  | 3.67 | 4.58 | 0.21 | 0.55 | 0.08 |
|  | Relative humidity |  |  |  | 1.00 | 1.00 | 0.12 |  |  |
| *Seoul, Korea* | | -3.68 | 10.36 | 0.72 |  |  |  |  |  |
|  | Temperature |  |  |  | 8.86 | 8.99 | <0.001 | 0.99 | 1.09 |
|  | Relative humidity |  |  |  | 8.99 | 9.00 | <0.001 |  |  |
| *Tokyo, Japan* | | 1.65 | 0.15 | 0.00 |  |  |  |  |  |
|  | Temperature |  |  |  | 4.88 | 5.80 | 0.01 | 0.93 | 0.78 |
|  | Relative humidity |  |  |  | 5.78 | 6.73 | 0.04 |  |  |
| *Kuala lumpur, Malaysia* | | -0.54 | 0.45 | 0.23 |  |  |  |  |  |
|  | Temperature |  |  |  | 8.10 | 8.54 | 0.00 | 1.00 | 0.21 |
|  | Relative humidity |  |  |  | 2.70 | 3.15 | 0.00 |  |  |
| **Lag 3d model** | |  |  |  |  |  |  |  |  |
| *Beijing, China* | | 1.43 | 0.11 | 0.00 |  |  |  |  |  |
|  | Temperature |  |  |  | 7.16 | 8.14 | <0.001 | 0.71 | 1.57 |
|  | Relative humidity |  |  |  | 1.94 | 2.40 | 0.41 |  |  |
| *Shanghai, China* | | 0.57 | 0.25 | 0.02 |  |  |  |  |  |
|  | Temperature |  |  |  | 1.00 | 1.00 | 0.19 | 0.91 | 0.08 |
|  | Relative humidity |  |  |  | 1.00 | 1.00 | 0.78 |  |  |
| *Wuhan, China* | | 4.66 | 0.09 | 0.00 |  |  |  |  |  |
|  | Temperature |  |  |  | 8.99 | 9.00 | 0.00 | 0.89 | 96.59 |
|  | Relative humidity |  |  |  | 8.95 | 9.00 | 0.00 |  |  |
| *Guangzhou, China* | | 0.01 | 0.38 | 0.98 |  |  |  |  |  |
|  | Temperature |  |  |  | 5.03 | 5.97 | 0.06 | 0.95 | 0.44 |
|  | Relative humidity |  |  |  | 7.56 | 8.36 | 0.10 |  |  |
| *Hong Kong, China* | | 0.74 | 0.10 | 7.23 |  |  |  |  |  |
|  | Temperature |  |  |  | 3.97 | 4.83 | 0.48 | 0.46 | 0.76 |
|  | Relative humidity |  |  |  | 2.30 | 2.82 | <0.001 |  |  |
| *Singapore* | | 1.32 | 0.08 | 0.00 |  |  |  |  |  |
|  | Temperature |  |  |  | 1.00 | 1.00 | 0.83 | 0.49 | 0.22 |
|  | Relative humidity |  |  |  | 1.00 | 1.00 | 0.13 |  |  |
| *Seoul, Korea* | | 2.04 | 0.35 | 0.00 |  |  |  |  |  |
|  | Temperature |  |  |  | 8.31 | 8.68 | 0.00 | 0.97 | 3.32 |
|  | Relative humidity |  |  |  | 8.88 | 8.99 | 0.00 |  |  |
| *Tokyo, Japan* | | 1.50 | 0.19 | 0.00 |  |  |  |  |  |
|  | Temperature |  |  |  | 6.82 | 7.73 | <0.001 | 0.91 | 0.98 |
|  | Relative humidity |  |  |  | 7.91 | 8.64 | <0.001 |  |  |
| *Kuala lumpur, Malaysia* | | -2.58 | 2.61 | 0.32 |  |  |  |  |  |
|  | Temperature |  |  |  | 6.22 | 6.48 | 0.38 | 1.00 | 0.07 |
|  | Relative humidity |  |  |  | 7.53 | 7.86 | 0.14 |  |  |
| **Lag 5d model** | |  |  |  |  |  |  |  |  |
| *Beijing, China* | | 1.37 | 0.12 | 0.00 |  |  |  |  |  |
|  | Temperature |  |  |  | 6.24 | 7.25 | 0.02 | 0.73 | 1.36 |
|  | Relative humidity |  |  |  | 6.65 | 7.74 | 0.00 |  |  |
| *Shanghai, China* | | 0.59 | 0.24 | 0.01 |  |  |  |  |  |
|  | Temperature |  |  |  | 1.31 | 1.56 | 0.13 | 0.92 | 0.07 |
|  | Relative humidity |  |  |  | 1.28 | 1.49 | 0.21 |  |  |
| *Wuhan, China* | | 0.27 | 0.36 | 0.44 |  |  |  |  |  |
|  | Temperature |  |  |  | 9.00 | 9.00 | 0.00 | 0.95 | 72.26 |
|  | Relative humidity |  |  |  | 9.00 | 9.00 | 0.00 |  |  |
| *Guangzhou, China* | | -0.15 | 0.37 | 0.70 |  |  |  |  |  |
|  | Temperature |  |  |  | 6.80 | 7.64 | 0.01 | 0.92 | 0.04 |
|  | Relative humidity |  |  |  | 8.70 | 8.94 | 0.01 |  |  |
| *Hong Kong, China* | | 0.71 | 0.11 | 0.00 |  |  |  |  |  |
|  | Temperature |  |  |  | 1.00 | 1.00 | 0.45 | 0.56 | 0.71 |
|  | Relative humidity |  |  |  | 3.83 | 4.79 | 0.04 |  |  |
| *Singapore* | | 1.34 | 0.08 | 0.00 |  |  |  |  |  |
|  | Temperature |  |  |  | 1.90 | 2.38 | 0.18 | 0.56 | 0.14 |
|  | Relative humidity |  |  |  | 1.00 | 1.00 | 0.16 |  |  |
| *Seoul, Korea* | | 2.53 | 0.21 | 0.00 |  |  |  |  |  |
|  | Temperature |  |  |  | 7.69 | 8.07 | 0.00 | 1.00 | 1.77 |
|  | Relative humidity |  |  |  | 9.00 | 9.00 | 0.00 |  |  |
| *Tokyo, Japan* | | 1.60 | 0.16 | 0.00 |  |  |  |  |  |
|  | Temperature |  |  |  | 8.79 | 8.97 | 0.00 | 0.87 | 0.89 |
|  | Relative humidity |  |  |  | 1.00 | 1.00 | 0.30 |  |  |
| *Kuala lumpur, Malaysia* | | -6.05 | 6.67 | 0.36 |  |  |  |  |  |
|  | Temperature |  |  |  | 1.00 | 1.00 | 0.07 | 1.00 | -0.12 |
|  | Relative humidity |  |  |  | 8.31 | 8.56 | 0.00 |  |  |
| **Lag 7d model** | |  |  |  |  |  |  |  |  |
| *Beijing, China* | | 1.31.40 | 0.12 | 0.00 |  |  |  |  |  |
|  | Temperature |  |  |  | 8.32 | 8.83 | 0.00 | 0.81 | 0.95 |
|  | Relative humidity |  |  |  | 8.61 | 8.94 | 0.00 |  |  |
| *Shanghai, China* | | 0.59 | 0.24 | 0.01 |  |  |  |  |  |
|  | Temperature |  |  |  | 1.00 | 1.00 | 0.38 | 0.90 | 0.11 |
|  | Relative humidity |  |  |  | 1.00 | 1.00 | 0.39 |  |  |
| *Wuhan, China* | | 4.27 | 0.09 | 0.00 |  |  |  |  |  |
|  | Temperature |  |  |  | 8.99 | 9.00 | 0.00 | 0.76 | 142.69 |
|  | Relative humidity |  |  |  | 8.99 | 9.00 | 0.00 |  |  |
| *Guangzhou, China* | | 0.11 | 0.34 | 0.75 |  |  |  |  |  |
|  | Temperature |  |  |  | 1.00 | 1.00 | 0.31 | 0.87 | 0.56 |
|  | Relative humidity |  |  |  | 4.46 | 5.47 | 0.05 |  |  |
| *Hong Kong, China* | | 0.75 | 0.10 | 0.00 |  |  |  |  |  |
|  | Temperature |  |  |  | 3.91 | 4.86 | 0.11 | 0.49 | 0.83 |
|  | Relative humidity |  |  |  | 1.00 | 1.00 | 0.85 |  |  |
| *Singapore* | | 1.40 | 0.08 | 0.00 |  |  |  |  |  |
|  | Temperature |  |  |  | 5.77 | 6.83 | <0.001 | 0.81 | 0.33 |
|  | Relative humidity |  |  |  | 1.75 | 2.20 | 0.64 |  |  |
| *Seoul, Korea* | | 2.57 | 0.18 | 0.00 |  |  |  |  |  |
|  | Temperature |  |  |  | 8.50 | 8.85 | 0.00 | 0.98 | 2.93 |
|  | Relative humidity |  |  |  | 8.09 | 8.45 | 0.00 |  |  |
| *Tokyo, Japan* | | 1.73 | 0.13 | 0.00 |  |  |  |  |  |
|  | Temperature |  |  |  | 4.95 | 5.96 | 0.07 | 0.86 | 1.21 |
|  | Relative humidity |  |  |  | 4.21 | 4.98 | <0.001 |  |  |
| *Kuala lumpur, Malaysia* | | -5.43 | 4.04 | 0.18 |  |  |  |  |  |
|  | Temperature |  |  |  | 5.87 | 6.20 | 0.03 | 1.00 | 0.04 |
|  | Relative humidity |  |  |  | 5.95 | 6.47 | 0.01 |  |  |
| **Lag 14d model** | |  |  |  |  |  |  |  |  |
| *Beijing, China* | | 1.23 | 0.13 | 0.00 |  |  |  |  |  |
|  | Temperature |  |  |  | 8.49 | 8.87 | 0.00 | 0.80 | 0.69 |
|  | Relative humidity |  |  |  | 5.98 | 6.97 | <0.001 |  |  |
| *Shanghai, China* | | 0.56 | 0.24 | 0.02 |  |  |  |  |  |
|  | Temperature |  |  |  | 2.40 | 3.00 | 0.56 | 0.93 | 0.02 |
|  | Relative humidity |  |  |  | 3.17 | 3.92 | 0.15 |  |  |
| *Wuhan, China* | | -0.52 | 1.05 | 0.62 |  |  |  |  |  |
|  | Temperature |  |  |  | 7.48 | 8.25 | 0.00 | 0.92 | 86.64 |
|  | Relative humidity |  |  |  | 9.00 | 9.00 | 0.00 |  |  |
| *Guangzhou, China* | | 0.15 | 0.32 | 0.64 |  |  |  |  |  |
|  | Temperature |  |  |  | 6.35 | 7.31 | 0.03 | 0.93 | 0.46 |
|  | Relative humidity |  |  |  | 1.00 | 1.00 | 0.23 |  |  |
| *Hong Kong, China* | | 0.74 | 0.10 | 0.00 |  |  |  |  |  |
|  | Temperature |  |  |  | 1.00 | 1.00 | 0.00 | 0.52 | 0.62 |
|  | Relative humidity |  |  |  | 1.00 | 1.00 | 0.00 |  |  |
| *Singapore* | | 1.65 | 0.07 | 0.00 |  |  |  |  |  |
|  | Temperature |  |  |  | 6.72 | 7.75 | 0.00 | 0.90 | 0.37 |
|  | Relative humidity |  |  |  | 1.00 | 1.00 | 0.10 |  |  |
| *Seoul, Korea* | | 2.63 | 0.17 | 0.00 |  |  |  |  |  |
|  | Temperature |  |  |  | 8.70 | 8.94 | 0.00 | 0.95 | 3.59 |
|  | Relative humidity |  |  |  | 8.65 | 8.98 | 0.00 |  |  |
| *Tokyo, Japan* | | 1.68 | 0.14 | 0.00 |  |  |  |  |  |
|  | Temperature |  |  |  | 2.31 | 2.87 | 0.10 | 0.90 | 0.74 |
|  | Relative humidity |  |  |  | 7.12 | 7.99 | 0.00 |  |  |
| *Kuala lumpur, Malaysia* | | -10.53 | 226.96 | 0.96 |  |  |  |  |  |
|  | Temperature |  |  |  | 8.02 | 8.03 | 0.08 | 1.00 | 0.09 |
|  | Relative humidity |  |  |  | 7.09 | 7.38 | 0.00 |  |  |

Edf: effective degrees of freedom; UBRE: Un-biased Risk Estimator
