# Supplementary figures and images for: The Influence of Average Temperature and Relative Humidity on New Cases of COVID-19: Time-Series Analysis
Source: JMIR Public Health Surveill. 2021 Jan 25;7(1):e20495. doi: 10.2196/20495 (PMC7836910; doi:10.2196/20495)

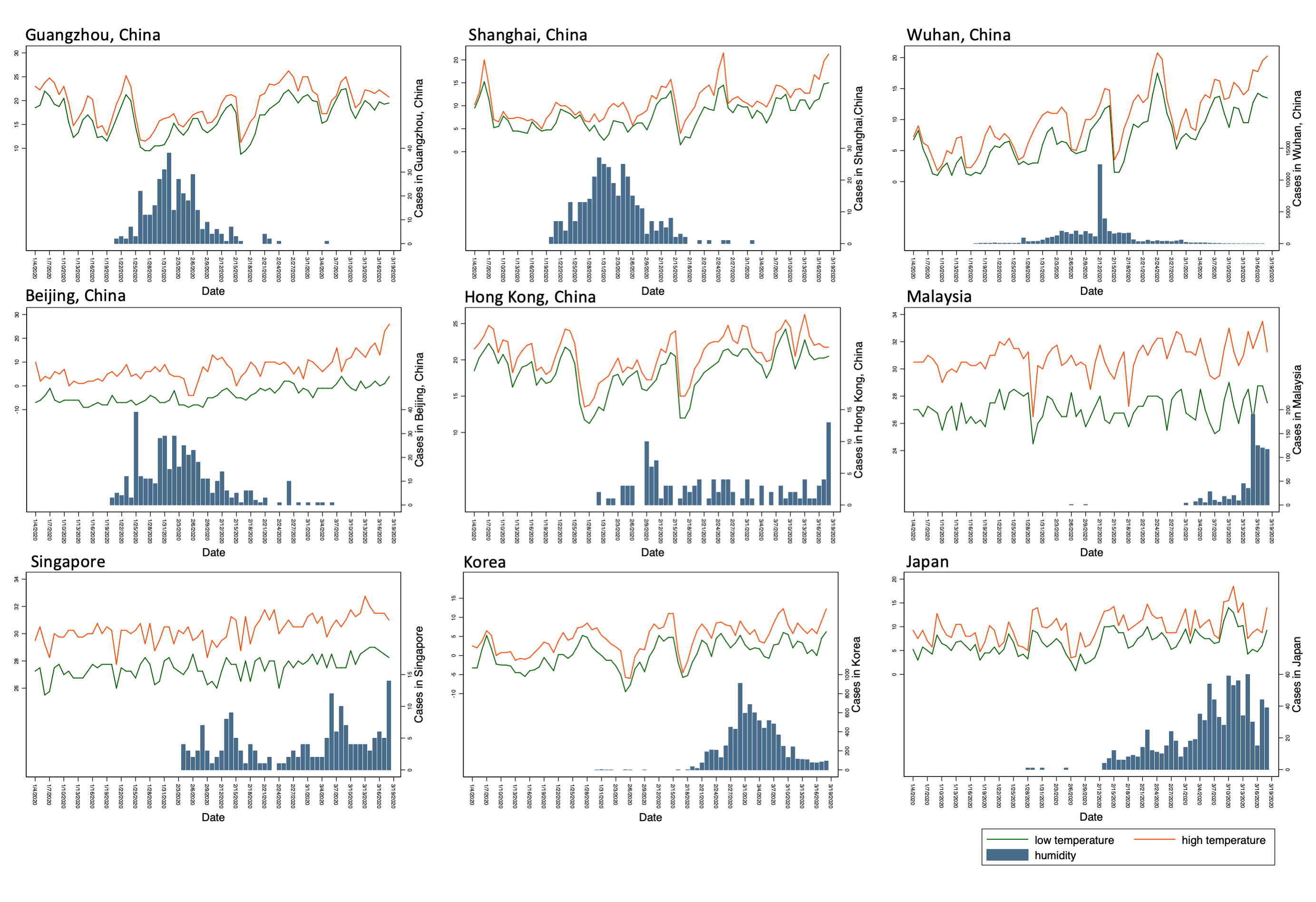

Supplement: Multimedia Appendix 2 [file publichealth_v7i1e20495_app2.png]

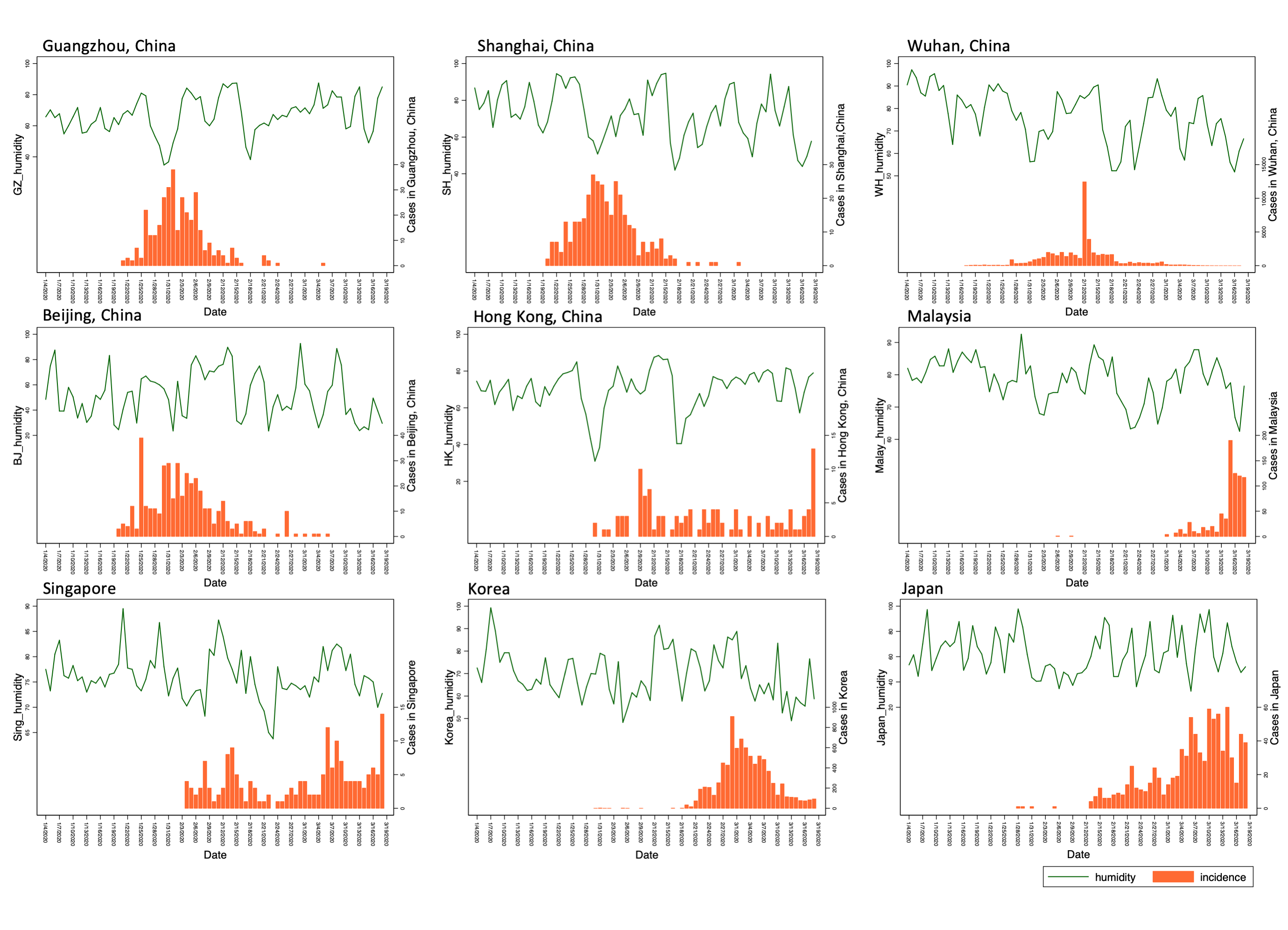

Supplement: Multimedia Appendix 3 [file publichealth_v7i1e20495_app3.png]

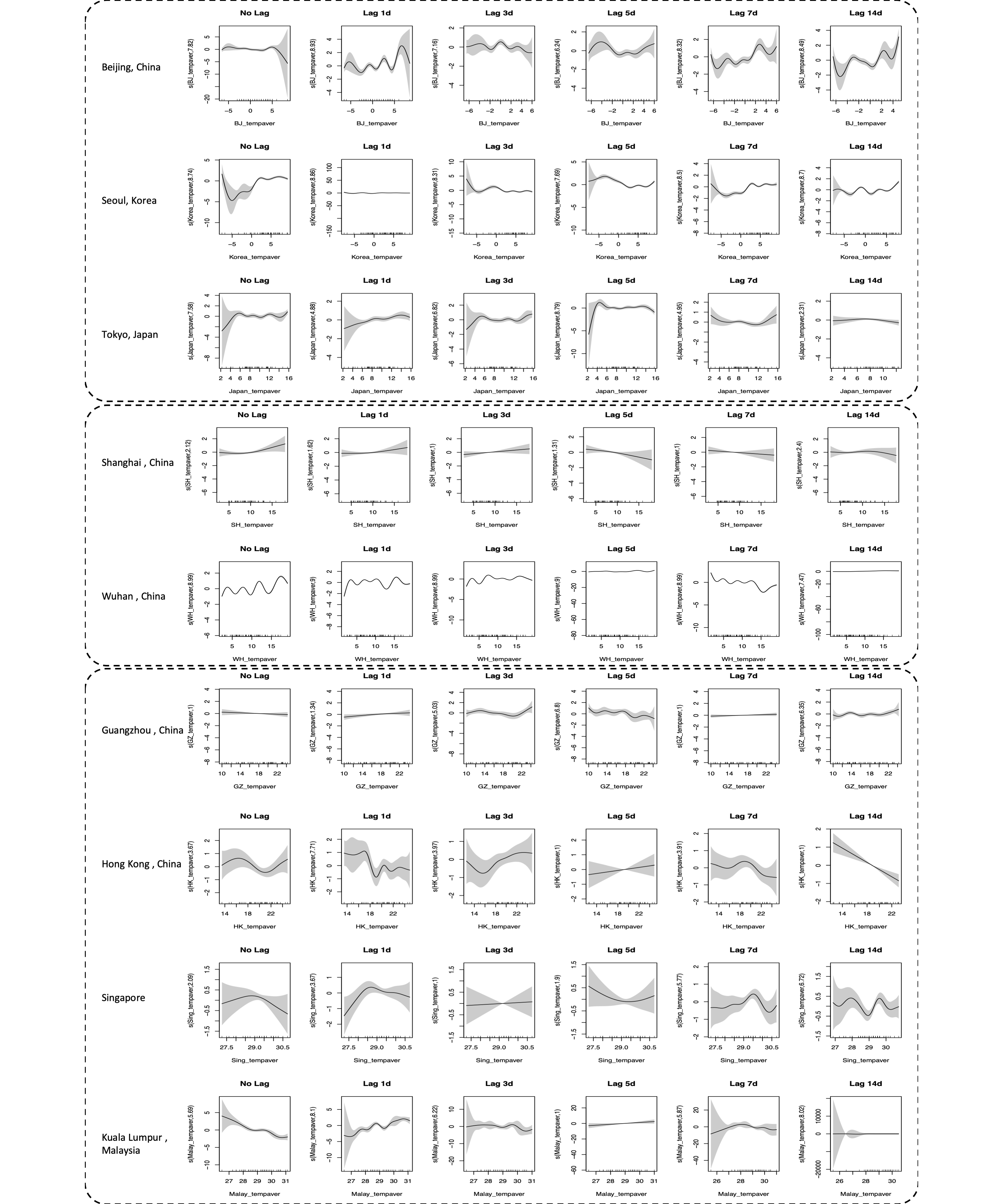

Supplement: Multimedia Appendix 4 [file publichealth_v7i1e20495_app4.png]

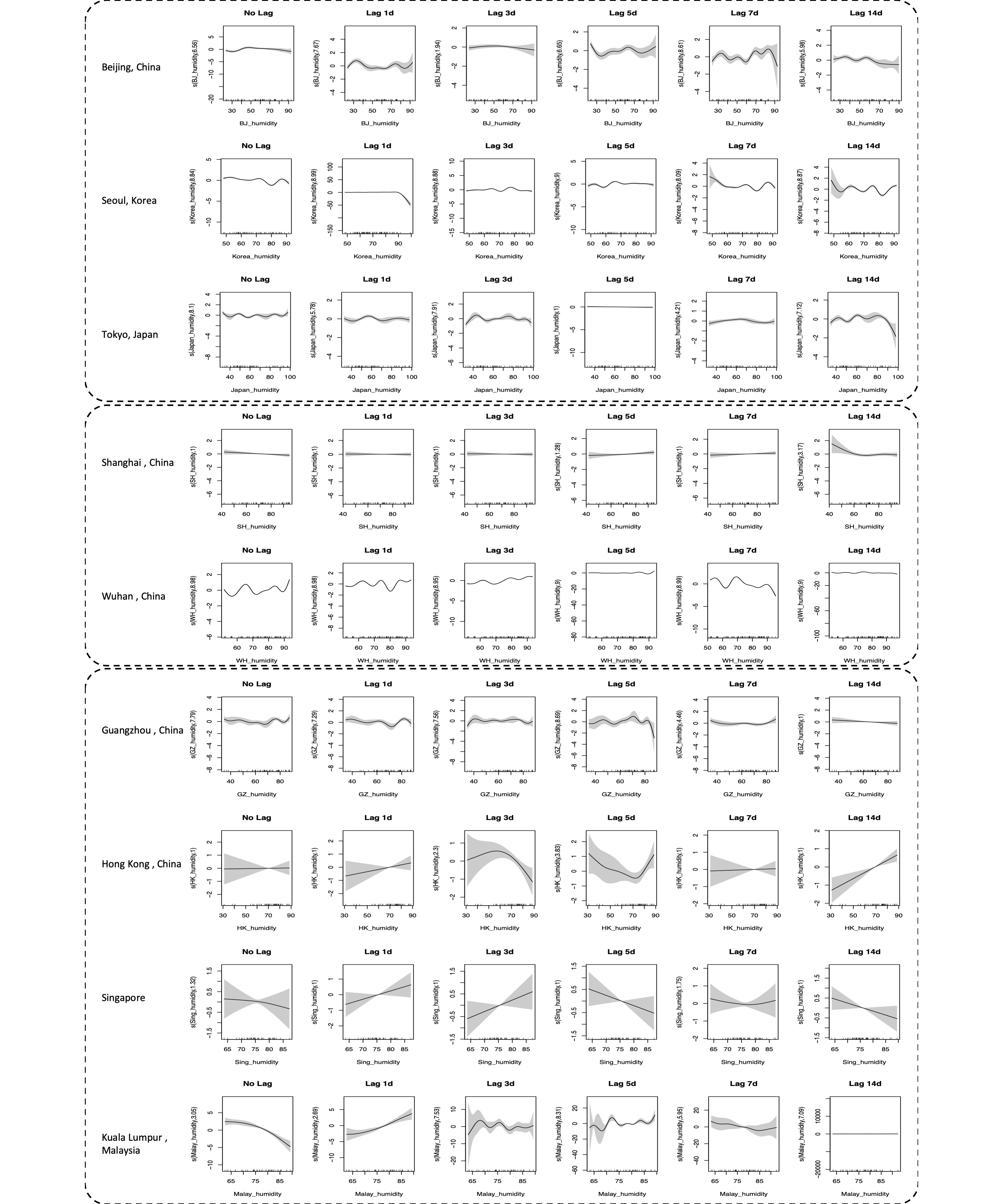

Supplement: Multimedia Appendix 5 [file publichealth_v7i1e20495_app5.png]
